# Occupational Burnout, Post‐Traumatic Stress Disorder and Intention to Resign Among Nursing Personnel During Public Health Emergencies

**DOI:** 10.1002/nop2.70555

**Published:** 2026-06-23

**Authors:** Jing Zhao, Leilei Wang, Yan Zhao, Jianing Gu, Yan Wu, Jingxun Chen, Xiangbo Meng

**Affiliations:** ^1^ Beijing Huilongguan Hospital Capital Medical University Beijing China; ^2^ Yanqing District Mental Health Hospital of Beijing Beijing China; ^3^ Beijing Yanqing District Hospital (Peking University Third Hospital Yanqing Hospital) Beijing China

**Keywords:** nursing personnel, occupational burnout, post‐traumatic stress disorder, public health emergencies, turnover intention

## Abstract

**Aim:**

Studies have shown that nursing staff are prone to post‐traumatic stress disorder (PTSD), occupational burnout and turnover tendency in public health emergencies, but the incidence of these three and their influencing factors during the outbreak of COVID‐19 have not been clarified. The aim of this study was to explore the mediating mechanism of occupational burnout between PTSD and turnover intention in nursing staff in public health emergencies.

**Design:**

The study was a cross‐sectional survey involving 1087 nursing personnels from 17 provinces in China. Convenience sampling was employed to select nursing personnel from frontline clinical settings in 17 Chinese provinces from March to May 2023. After excluding 13 invalid questionnaires, 1087 valid questionnaires were obtained, resulting in an overall validity rate of 98.8%.

**Methods:**

Civilian version of Post‐traumatic Stress Disorder Questionnaire (PCL‐C), Maslach Burnout Inventory‐General Survey (MBI‐GS) and Turnover Intention Questionnaire (TIQ) were used respectively. Post‐traumatic stress symptoms, occupational burnout and turnover tendency were assessed. Ethical review approval: Approval unit: Medical Ethics Committee of Beijing Huilongguan Hospital; Approval number: 2023‐23—Science and Technology.

**Results:**

15.8% of nursing personnel had PTSD, 71.8% had occupational burnout and 63.7% had turnover intention. The scores of occupational burnout and turnover intention of nursing personnel with PTSD were higher than those without PTSD. Each dimension of PTSD was positively correlated with all dimensions of occupational burnout and nurse resignation (all *p* < 0.001). Occupational burnout was also positively correlated with all dimensions of turnover intention of nursing personnel (*p* < 0.001). Occupational burnout partially mediated the relationship between PTSD and turnover intention of nursing personnel in COVID‐19.

**Conclusions:**

During public health emergencies, during the COVID‐19 pandemic, nursing personnel had PTSD, occupational burnout and turnover intention. PTSD plays a significant direct and indirect role in nurse turnover. Therefore, it should be a standard practice to monitor and manage PTSD among nursing personnel and provide proactive support systems in order to retain personnel and promote the best nursing practice standards during public health emergencies.

**Patient or Public Contribution:**

No patient or public contribution.

## 1 Introduction

Globally, public health events, including infectious disease outbreaks and natural disasters, often have significant impacts on healthcare systems. As integral components of the healthcare system, nursing personnel play a crucial role in public health events (Bakhamis et al. 2019). However, during the outbreak of COVID‐19, in the context of China, they frequently face the risk of occupational burnout owing to their high work intensity, uncertain working environments and responsibility for patients' well‐being (Jing et al. 2025). Occupational burnout not only affects the job performance and quality of life of nursing personnel, but may also be associated with more severe mental health issues such as PTSD (Wu et al. 2020).

PTSD is a psychological disorder that occurs after a traumatic event and is characterised by persistent fear, stressful memories and avoidance of trauma‐related stimuli, among other symptoms (El‐Hage et al. 2020). During public health emergencies, nursing personnel often directly experience or witness trauma‐related situations involving patients, which can lead to the development of PTSD symptoms after the event. Studies have found that, during the COVID‐19 pandemic, the detection rate of PTSD among frontline nursing personnel was as high as 44.86% (Hu et al. 2020), while other healthcare workers had a rate of only 13.99% (Fan et al. 2020). Occupational burnout may serve as a mediation variable, further exacerbating mental health issues faced by nursing personnel (Zasiekina and Martyniuk 2025).

In addition to mental health issues, the impact of public health events on the intention to resign among nursing personnel has been studied (Johnson and O'Rourke 2020; Smith et al. 2006; Zhang and Liu 2020). Owing to increased job stress and psychological burden, nursing personnel may contemplate leaving their current work environment in search of better career prospects or improved mental well‐being. According to Smith et al. (2006), nursing personnel experience a decrease in job satisfaction and an increase in the intention to resign when facing sudden public health events such as influenza pandemics. Another study found a significant rise in the intention to resign among nursing personnel during the COVID‐19 pandemic compared to before (Zhang and Liu 2020).

Therefore, understanding the relationship between occupational burnout, PTSD and the turnover intention is important for implementing appropriate interventions, maintaining the psychological well‐being of nursing personnel and enhancing job satisfaction (Jing et al. 2025). This is especially crucial during the COVID‐19 pandemic, when nursing personnel experience a significant increase in job stress and workload, which may further elevate resignation and burnout rates. Further research is needed to investigate the prevalence, associated factors and their relationships with PTSD.

Similar to most studies that predominantly focus on developed countries or regions, there exists a gap in understanding the experiences of nursing professionals in emergency public health events within the context of China. Given the influence of traditional Chinese culture, the experiences of nursing personnel during emergencies may differ. Different types of healthcare institutions and work environments can also have varying impacts on the occupational burnout and mental health of nursing professionals. In addition, the current research mainly focuses on the early stage of the outbreak of COVID‐19 and there is a lack of investigation on the mental health status and occupational burnout of nursing personnel in the post‐epidemic era. Our aim is to investigate, in the Chinese context, the mediation mechanisms between occupational burnout, PTSD following occupational trauma and the turnover intention among nursing personnel in the face of public health emergencies.

## 2 Design

The study was a cross‐sectional survey involving 1087 nursing personnel from 17 provinces in China. Convenience sampling was employed to select nursing personnel from frontline clinical settings in 17 Chinese provinces from March to May 2023. The inclusion criteria for the study population were registered nurses with practical qualifications, more than one year of clinical experience, had been employed since the beginning of the COVID‐19 pandemic (especially since January 2023) and voluntary participation in the study. Participants who have not completed the answers, and answered the questions for too short a time (< 5 min) were excluded. Participants were invited to complete a series of online questionnaires via the Wenjuanxing platform (https://www.wjx.cn), which was distributed via Wechat and all the participants carefully read an informed consent form and willingly participated in the study. Informed consent was obtained electronically and all participants' information remained anonymous. After completing the questionnaires, 1100 participants were included in the study. After excluding 13 invalid questionnaires, 1087 valid questionnaires were obtained, resulting in an overall validity rate of 98.8%. This study was approved by the Ethics Committee of Medical Ethics Committee of Beijing Huilongguan Hospital, with approval number: 2023–23—Science and Technology.

## 3 Methods

### 3.1 Clinical Evaluation

The trauma experience of nursing personnel was assessed using the Civilian Version of the PTSD Checklist (PCL‐C) developed by the United States PTSD Research Center. This checklist comprised 17 items and was administered in a self‐reporting format. It is divided into three sections: re‐experiencing symptoms (items 1–5), PTSD emotional numbing and avoidance symptoms (items 6–12) and irritability symptoms caused by hyperarousal (items 13–17). Each item on the checklist is rated on a 5‐point scale ranging from 1 (not at all) to 5 (extremely severe). Higher scores indicate a higher likelihood of developing PTSD. Research has shown that a total score > 38 is considered positive for PTSD (Zhao et al. 2021). The Chinese version of the PCL‐C has Cronbach's *α* coefficients ranging from 0.88 to 0.94 and test–retest reliability ranging from 0.83 to 0.88 (Chen et al. 2005).

The Maslach Burnout Inventory‐General Survey (MBI‐GS) was developed by Maslach and Jackson to assess the level of occupational burnout (Maslach and Jackson 1986). The Chinese version of the MBI‐GS has been revised by Chaoping Li and colleagues (Li and Shi 2003) and has been shown to have good reliability and validity. It comprises three dimensions: emotional exhaustion (items 1–5), cynicism (items 6–9) and low personal achievement (items 10–15). It is scored on a 7‐point Likert scale ranging from 0 (never) to 6 (very often). Higher scores indicate higher levels of occupational burnout (Huang et al. 2021). Cronbach's α coefficient for the scale was 0.829.

The Turnover Intention Questionnaire (TIQ) is used to assess the participants' intentions to resign. The questionnaire consisted of three dimensions and six items: Items 1 and 6 comprised Dimension I, indicating the likelihood of leaving the current job; Items 2 and 3 comprised Dimension II, indicating the likelihood of seeking other employment; and Items 4 and 5 comprised Dimension III, indicating the likelihood of obtaining other employment. The questionnaire uses a 4‐point Likert scale, with reverse scoring for items 1 to 4, with higher scores indicating a stronger intention to resign. The Cronbach's *α* coefficient for the scale is 0.773, and it has been widely used among healthcare personnel (BowenXue et al. 2024).

### 3.2 Statistical Method

SPSS software (version 23.0) was used for the data analysis. Frequency and percentage were used for descriptive statistics, and for data following a normal distribution represented as mean ± standard deviations. A chi‐squared test was used to analyse the categorical variables, while a *t*‐test was used to analyse continuous variables. Pearson's correlation analysis was used. The level of significance was set at *p* < 0.05. The bootstrap method was used to analyse the significance of the mediating effect of occupational burnout on the relationship between total PTSD scores and nurse turnover, with a sample size of 5000 and a 95% confidence interval.

## 4 Results

### 4.1 Sociodemographic Characteristics Associated With PTSD, Occupational Burnout and Turnover Intention

As can be seen in Table 1, 1087 participants had an average age of 35.95 (±8.67) years and were assessed to have worked for 14.53 (±9.68) years. There were 225 males (20.7%) and 862 females (79.3%). This study indicates that 15.8% of nursing personnel had PTSD, 71.8% had occupational burnout and 63.7% had turnover intention. The proportion of male nursing personnel experiencing PTSD and occupational burnout was higher than that of female nursing personnel (*p* < 0.01). The more serious the concern about oneself or family members being infected with COVID‐19, the higher the proportion of nursing personnel experiencing PTSD and turnover intention (*p* < 0.01). As the level of stress to life or work increased for COVID‐19, the proportion of nursing personnel experiencing PTSD, occupational burnout and turnover intention increased significantly (*p* < 0.01). Among nursing personnel in hospitals below the secondary level, the proportion of occupational burnout was the highest, followed by those in secondary and tertiary hospitals (*p* < 0.01). Among nursing personnel in Beijing, the proportion of PTSD and turnover intention was significantly higher than that in other regions (*p* < 0.01). As Table 2 showed among nursing personnel with intermediate and higher professional titles, the proportion of PTSD and turnover intention was significantly higher than that among nursing personnel with intermediate and higher professional titles (*p* < 0.01). Compared to female nurses and nurse practitioners, male nurses and intermediate or above titles nurses had higher scores for PTSD, occupational burnout and turnover intention (all *p*'s < 0.05).

**TABLE 1:** Table of general demographic variables, machine PTSD, occupational burnout and turnover intention.

Variables	*n*	%	With PTSD			Have burnout			There are nurses leaving		
			*n*	%	*p*	*n*	%	*p*	*n*	%	*p*
Gender					< 0.001			0.001			0.335
Male	225	20.7	54	24.0		181	80.4		148	65.8	
Female	862	79.3	118	13.7		599	69.5		537	62.3	
Are you worried that you or a family member may contract COVID‐19?					< 0.001			0.055			< 0.001
Never worry	84	7.7	6	7.1		60	71.4		34	40.5	
Occasional worries	249	22.9	19	7.6		173	69.5		138	55.4	
Sometimes worry	329	30.3	31	9.4		221	67.2		207	62.9	
Frequent worries	237	21.8	50	21.1		183	77.2		164	69.2	
Always worried	188	17.3	66	35.1		143	76.1		142	75.5	
Do you feel that the current new coronary pneumonia has added stress to your life or work?					< 0.001			< 0.001			< 0.001
No	112	10.3	4	3.6		74	66.1		39	34.8	
Mild	357	32.8	12	3.4		237	66.4		181	50.7	
Moderate	375	34.5	56	14.9		269	71.7		264	70.4	
Severe	171	15.7	61	35.7		146	85.4		141	82.5	
Extremely heavy	72	6.6	39	54.2		54	75		60	83.3	
Level of hospital					0.241			< 0.001			0.099
Grade 3	837	77.0	130	15.5		574	68.6		529	63.2	
Grade 2	138	12.7	19	13.8		107	77.5		94	68.1	
Below the second level	112	10.3	23	20.5		99	88.4		62	55.4	
Beijing					< 0.001			0.339			< 0.001
Yes	763	70.2	141	18.5		554	72.6		521	68.3	
No	324	29.8	31	9.6		226	69.8		164	50.6	
Title					0.020			0.678			< 0.001
Intermediate and above	452	41.6	88	19.5		330	73.0		318	70.4	
Nurse practitioner	635	58.4	84	13.2		450	70.9		367	57.8	

**TABLE 2:** Differences in gender and title on the dimensions of PTSD, occupational burnout and turnover intention.

Variable	Gender		*t*	*p*	Title		*t*	*p*
	Male	Female			Intermediate and above	Nurse practitioner		
Re‐experiencing symptoms	8.87 ± 4.61	7.79 ± 3.59	3.256	0.001	8.57 ± 3.97	7.61 ± 3.71	4.042	< 0.001
Avoidance and numbing symptoms	12.35 ± 6.35	10.65 ± 4.95	3.725	< 0.001	11.66 ± 5.43	10.54 ± 5.19	3.451	0.001
Hyperarousal symptoms	9.71 ± 4.95	8.91 ± 4.21	2.224	0.027	9.65 ± 4.40	8.66 ± 4.33	3.677	< 0.001
PTSD total score	30.92 ± 15.31	27.35 ± 12.00	3.252	0.001	29.88 ± 13.01	26.81 ± 12.56	3.913	< 0.001
Emotional exhaustion	9.08 ± 8.33	7.96 ± 7.24	1.839	0.067	10.02 ± 7.58	6.89 ± 7.15	6.927	< 0.001
Cynicism	6.26 ± 6.34	4.51 ± 5.11	3.828	< 0.001	5.79 ± 5.44	4.21 ± 5.33	4.785	< 0.001
Low personal accomplishment	20.60 ± 10.44	18.91 ± 10.41	2.166	0.031	18.67 ± 9.57	19.68 ± 11.01	−1.605	0.109
Burnout total score	35.93 ± 16.87	31.34 ± 15.27	3.927	< 0.001	34.48 ± 15.57	30.73 ± 15.65	3.9	< 0.001
TIQ Dimension I	3.50 ± 1.60	3.15 ± 1.44	2.997	0.003	3.36 ± 1.45	3.12 ± 1.49	2.598	0.009
TIQ Dimension II	3.70 ± 1.70	3.46 ± 1.57	2.012	0.045	3.67 ± 1.59	3.40 ± 1.60	2.849	0.004
TIQ Dimension III	3.61 ± 1.70	3.27 ± 1.45	2.752	0.006	3.46 ± 1.47	3.26 ± 1.54	2.116	0.035
TIQ total score	10.81 ± 4.71	9.88 ± 4.13	2.715	0.007	10.49 ± 4.14	9.78 ± 4.34	2.719	0.007

Abbreviations: PTSD, post‐traumatic stress disorder; TIQ, Turnover Intention Questionnaire.

Table 3 displays differences in the presence or absence of PTSD on the dimensions of age, years of work experience, occupational burnout and turnover intention. The impact of PTSD on the occupational burnout and turnover intention of nursing personnel Compared to nursing personnel without PTSD, those with PTSD have higher scores in terms of burnout emotional exhaustion, feelings of cynicism, low personal achievement, the likelihood of current job resignation (TIQ Dimension I), seeking other employment (TIQ Dimension II), obtaining other employment (TIQ Dimension III) and the overall turnover intention questionnaire score.

**TABLE 3:** Differences in the presence or absence of PTSD on the dimensions of age, years of work experience, occupational burnout and turnover intention.

	No PTSD (915)	With PTSD (172)	*t*	*p*
Burnout emotional exhaustion	6.30 ± 5.86	18.27 ± 7.18	−20.619	< 0.001
Feeling of cynicism	3.51 ± 4.11	12.10 ± 5.89	−18.307	< 0.001
Low personal achievement	19.40 ± 10.92	18.50 ± 7.37	1.353	0.177
Burnout total score	29.21 ± 14.10	48.70 ± 13.61	−17.129	< 0.001
TIQ Dimension I	2.99 ± 1.30	4.50 ± 1.70	−11.081	< 0.001
TIQ Dimension II	3.28 ± 1.47	4.73 ± 1.70	−10.500	< 0.001
TIQ Dimension III	3.12 ± 1.35	4.51 ± 1.77	−9.373	< 0.001
Nurse separation parity	1.56 ± 0.63	2.29 ± 0.80	−11.200	< 0.001
Age	35.40 ± 8.53	38.90 ± 8.78	−4.92	< 0.001
Years of work	13.88 ± 9.56	18.00 ± 9.63	−5.176	< 0.001

Abbreviations: PTSD, post‐traumatic stress disorder; TIQ, Turnover Intention Questionnaire.

### 4.2 The Relationship Between PTSD Among Nursing Personnel and Occupational Burnout as Well as Turnover Intention

**FIGURE 1:**
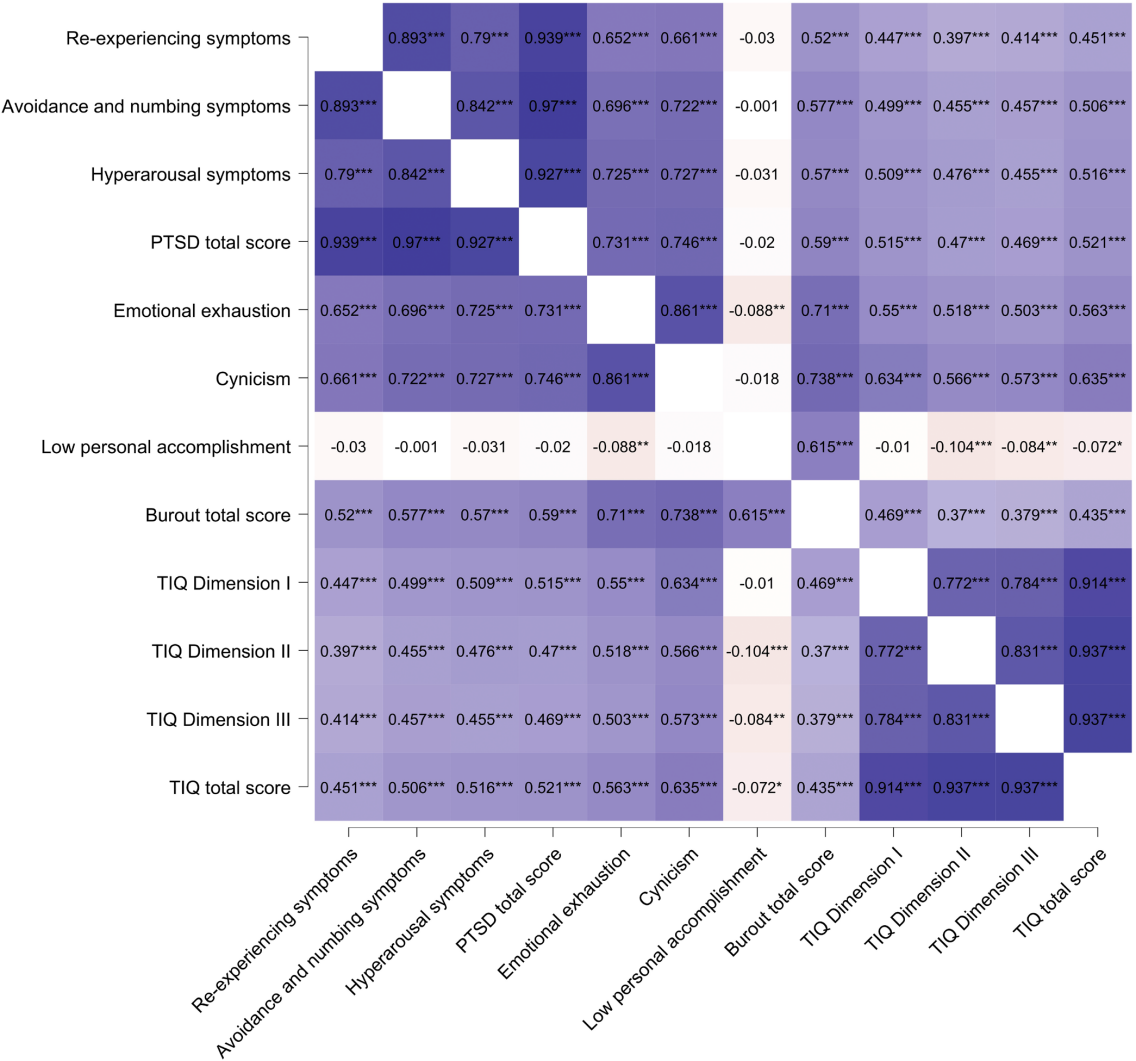
Correlation analysis of PTSD, burnout and propensity to leave among nursing personnel. *p < 0.05, **p < 0.01, ***p < 0.001.

As shown in Figure 1, the results of the correlation analysis indicate that each dimension of PTSD is positively correlated all other dimensions of occupational burnout and turnover intention (*p* < 0.001). Each dimension of PTSD is positively correlated with each dimension of nurse turnover intention (*p* < 0.001). The dimensions of nurse turnover intention are negatively correlated with the high achievement dimension of occupational burnout (*p* < 0.001) and positively correlated with all other dimensions (*p* < 0.001).

### 4.3 Mediation Effect Between PTSD Total Score, Occupational Burnout and Nurse Turnover

The total PTSD score was used as the independent variable, occupational burnout as the mediating variable and nurse turnover as the dependent variable. The ‘PROCESS procedure’ developed by Hayes was employed for mediation analysis. To account for potential confounding effects, we included age, gender, years of work, title, level of hospital, location and fear of contracting COVID‐19 for nurses and their families as covariates in our statistical models to control for their influence on the primary outcomes. Bootstrap resampling with 5000 samples and a 95% confidence interval was used to assess the significance of the mediation effect of occupational burnout on the relationship between the total PTSD score and nurse turnover.

**FIGURE 2:**
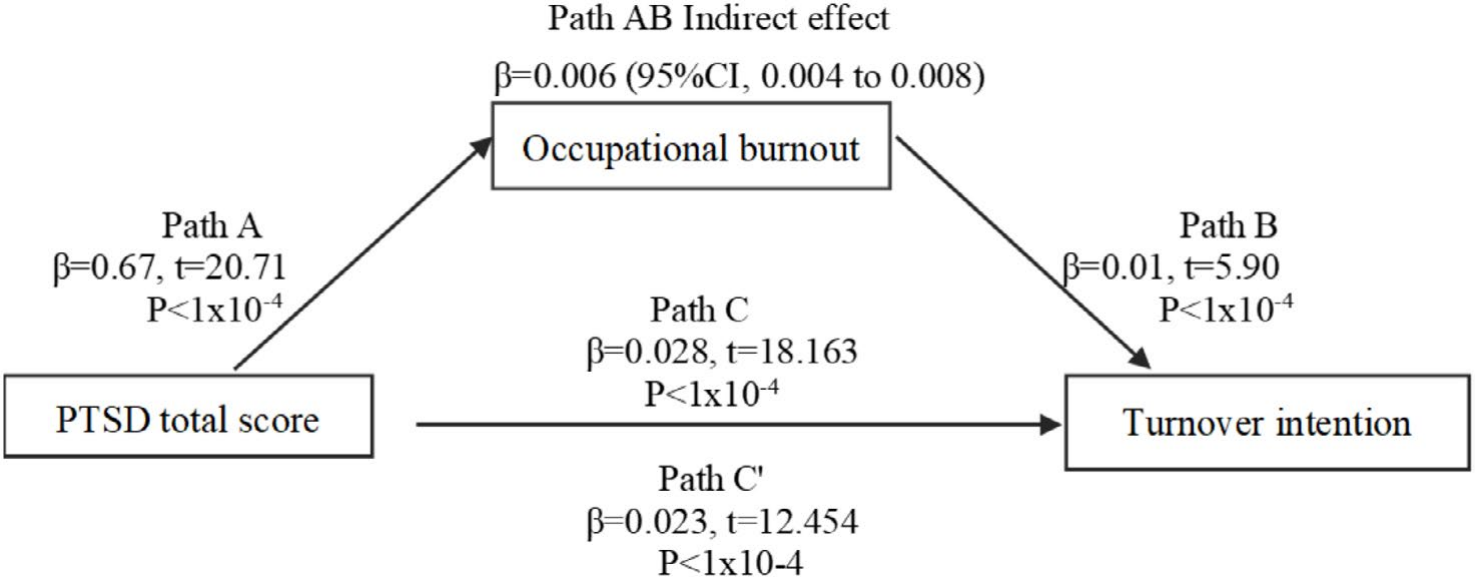
Path diagram of the mediation model (*X* = PTSD total score; *Y* = turnover intention; occupational burnout as mediators). Path C′ and Path C represent the direct and total effect between PTSD total score and turnover intention. Path AB represents the mediation effect and is significant at *p* < 0.05 in the models.

As shown in Figure 2, the path does not include zero; it indicates a significant mediation effect of the mediation variable. The results suggested that the total PTSD score could directly predict nurse turnover, with a direct effect value of 0.023 and an indirect effect value of 0.006; intermediary effects accounted for 21.43%.

## 5 Discussion

This study assessed the status and factors influencing occupational burnout, PTSD and turnover intention of nursing personnel during public health emergencies. We also analysed the relationship between these occupational burnout dimensions and PTSD and their impact on turnover intention. Based on existing data, this study identified the mediating role of occupational burnout in the relationship between PTSD and turnover intention.

Our study found that a high proportion of nursing personnel experienced PTSD, occupational burnout and turnover intention. Starting from January 8, 2023, the Chinese government reclassified COVID‐19 as a Class B notifiable infectious disease. Adjustments were made to the prevention and control policies and measures. During the research period from March to May 2023, COVID‐19 was in a widespread pandemic stage in China. Consequently, a survey was conducted on the research subjects during this phase. The COVID‐19 pandemic has placed immense stress on healthcare systems worldwide; the number of infections increased sharply and the work pressure increased, leading to the nurses' sense of occupational burnout intensifying. Chinese culture emphasises family responsibilities, and nurses find it difficult to juggle family in high‐pressure jobs; this conflict may lead them to choose to leave their jobs to return to their families (Zhou et al. 2018; Yang et al. 2020). Nurses worry about transmitting the virus to their families, and the psychological burden may prompt them to leave their jobs to protect their families. Prolonged working hours, emotional challenges and witnessing patients' suffering and death all contribute to psychological distress. The accumulation of stress coupled with the fear of infection can exacerbate the symptoms of PTSD (Liu et al. 2023) and the desire to leave the nursing profession (World Health Organization 2020).

Chinese culture emphasises collective interests, and nurses may neglect individual psychological needs due to a strong sense of responsibility and repress emotions for a long time, increasing the risk of PTSD (Zhang 2021). In addition, Chinese people are reserved and introverted and there is a sense of stigma for psychological problems (Tu and Zhang 2022). Chinese culture does not encourage open expression of emotions, so it may be difficult for nurses to seek psychological support, resulting in the trauma experience could not be resolved in time (Fang and Zhang 2020). During the pandemic period, the healthcare system was under tremendous pressure, often resulting in inadequate support systems for nursing personnel. Without sufficient support, nursing personnel may struggle to manage their stress levels, thereby increasing their likelihood of developing PTSD and occupational burnout (Kang 2022).

Research indicates that during public health emergencies, male nursing personnel have higher rates of PTSD and occupational burnout and the severity of trauma symptoms, occupational burnout and turnover tendency are more serious. Possible reasons for this could include the significant role played by Chinese sociocultural factors in both male and female nursing personnel. Nursing remains a predominantly female‐dominated profession, and male nursing personnel in this field may face unique sex‐related challenges such as role expectations and societal pressures. These factors might lead to increased stress and feelings of isolation among male nursing personnel, reduced job satisfaction and an elevated risk of developing both PTSD and occupational burnout (Baires et al. 2023; Evans et al. 2020). Additionally, males and females tend to have different coping mechanisms and ways of expressing emotions, which can influence their responses to the stress of public health emergencies. Studies suggest that males may be less inclined to seek help proactively or openly express their emotions, potentially leading to the internalisation of stress and adverse psychological outcomes (Chen, Pierson, et al. 2021; Wong et al. 2018). Male nurses may face greater financial stress, especially in cases of income instability or inadequate benefits during the pandemic, which may exacerbate their burnout and turnover tendency (Deng et al. 2024). Faced with the increase in the number of infected people and heavy work tasks, male nurses are usually assigned to more physically demanding work tasks, arranged to high‐risk positions and are more likely to feel physical fatigue and occupational burnout, which intensifies the turnover tendency (Foster et al. 2024).

Nursing personnel with prolonged careers and older age may experience a greater accumulation of occupational stress and workload, which could make them more vulnerable to public health emergencies. With age, additional stress from personal life, family and other sources can increase, further adding to their psychological burden. In community hospitals, nursing personnel may face the most severe occupational burnout because these hospitals often have limited resources for dealing with high work pressures and loads, especially in the context of COVID‐19. Research has shown that community hospitals often lack adequate staffing, equipment and training support, leading to excessive workloads for nurses (Charlwood et al. 2024; Kim and Bae 2014). Some community hospitals may have imperfect management and operational mechanisms, and lack effective communication and decision‐making processes, which can make nurses feel unable to cope with work stress, thereby increasing the risk of occupational burnout (Dall'Ora et al. 2020). The higher the professional title, the more serious the PTSD, occupational burnout and turnover tendency. Nurses with high professional titles usually take on more management and decision‐making responsibilities, coordinate resources, develop care plans and ensure implementation in the face of COVID‐19 and mentor nurses with low professional titles. This responsibility and stress and high‐intensity work increase the psychological burden, which can easily lead to PTSD and burnout (Zhang et al. 2024; Chen, Wang, et al. 2021). Nurses with high professional titles often face critically ill patients and their families, frequently experience life and death scenes and have huge emotional consumption, which can easily cause PTSD and emotional numbness and then have the idea of quitting (Li et al. 2021).

Nursing personnel in Beijing face demanding job requirements, including long hours wearing protective gear, rapid response to emergencies and exposure to high‐risk infections. These factors increase nurses' psychological burden and may make them more susceptible to symptoms of PTSD, increasing their tendency to leave the profession. Nursing personnel with intermediate or higher professional titles often bear more job responsibilities and management duties, which require them to face greater work pressures and loads. During COVID‐19, due to the surge in patient numbers and shortage of medical resources, they may face even greater workloads and time pressures, increasing the risk of developing PTSD and turnover intention (Kelly et al. 2019). Mid‐level and higher‐level nurses typically have more extensive clinical experience and professional knowledge; however, during the pandemic, they may face challenges beyond their training and experience. This can lead to feelings of helplessness and frustration, further increasing the risk of PTSD and turnover intention (Nantsupawat et al. 2024).

In terms of occupational burnout, nurses with PTSD exhibited higher levels of emotional exhaustion and depersonalisation. This may reflect the negative impact of PTSD on emotion regulation and coping abilities, causing nursing personnel to struggle with the emotional demands of their work. This is consistent with the findings of Ling et al. (2020), who identified emotional exhaustion and depersonalisation as core components of occupational burnout (Ling et al. 2020). Furthermore, nursing personnel with PTSD scored higher on turnover intentions. They may consider leaving their current jobs, seeking other employment or obtaining alternative positions. This could be because nursing personnel with PTSD feel pressure and discomfort in their work environment and wish to escape from an environment that may trigger traumatic memories. This aligns with the findings of Chipps et al. (2024) who observed a correlation between maladaptation to the work environment, decreased job satisfaction and nurses' intentions to leave their positions (Chipps et al. 2024). In terms of individual characteristics, nursing personnel with PTSD had a higher average age and more work experience. This is consistent with the findings of Creamer et al. (2005), who suggested that age and experience may be related to PTSD incidence (Creamer et al. 2005).

Our study found that in the post‐COVID‐19 era, nursing staff had a high proportion of PTSD, occupational burnout and turnover tendency, and the correlation between them was significant. These findings not only revealed that nursing staff were prone to severe mental health problems during public health emergencies, but also showed significant problems in their working status. Medical institutions should pay attention to the mental health of nursing staff, establish a support system, reduce work pressure and pay attention to the burnout of staff so as to reduce the turnover rate. For nursing staff themselves, when there are turnover tendency thoughts and occupational burnout, they should improve their mental health awareness and actively seek support and help. In addition, according to the results of the mediation effect analysis, occupational burnout plays an intermediary role in PTSD and turnover intention, which suggests that the intervention of occupational burnout may be a way to reduce the turnover intention caused by PTSD. However, we could not ignore the influence of other problems on turnover intention, such as insomnia, anxiety and depression symptoms caused by PTSD, income and family–work conflict, which are all worthy of our further consideration.

We acknowledge certain limitations of our study, owing to sample collection constraints and the use of a cross‐sectional research design. The use of convenience sampling, while practical, may introduce selection bias. The reliance on self‐reported online questionnaires may introduce response bias, particularly given the sensitive nature of the questions. Implementing anonymity, incentive measures, shortening survey time, simplifying questions and pre‐testing could potentially mitigate these biases. We recognise that cross‐sectional studies limit us from making causal inferences, and our current research may not have revealed the complete mechanism by which stress disorders among nursing personnel affect nurse turnover. While we observed associations between PTSD and occupational burnout, turnover intention, age and years of work in the existing data, we did not examine other potential variables and pathways. In addition, we did not discuss the mediation or moderate effects of availability of mental health resources, job autonomy, organisational support and work shifts, although previous studies have found that these factors are helpful to improve occupational burnout and turnover intention (Gherman et al. 2022; Shu et al. 2022). Other studies have found that quality of professional life, the interplay of burnout, secondary traumatic stress, compassion fatigue and compassion satisfaction. The interplay of burnout, secondary traumatic stress, compassion fatigue and compassion satisfaction (Chlan 2025; Zasiekina and Martyniuk 2025).

Further research is required for a more comprehensive understanding of this issue. Future research should explore the impact of stress disorders on nurse turnover among nursing personnel through various avenues. Different research designs, such as longitudinal studies and mixed‐methods research, can provide additional insights. Longitudinal studies can help track changes in nurses over time and provide a better understanding of the dynamic relationship between stress disorders and turnover. The mixed method research can combine quantitative and qualitative data, and adopt questionnaire survey and face‐to‐face interview to conduct detailed mental health status assessment for nurses with turnover tendency, so as to gain a deeper understanding of nurses' experiences and coping strategies in the face of stress disorder and how these strategies affect their decisions to leave their positions. Furthermore, expanding the scope of the sample and employing different research methods are essential. By encompassing a more diverse range of nursing personnel with varying backgrounds and characteristics, more representative conclusions can be drawn. Additionally, cross‐validation of multiple research methods could enhance our understanding of the pathways through which stress disorders among nursing personnel affect turnover, thereby ensuring the robustness and reliability of our findings.

## 6 Conclusions

The research findings indicate that PTSD exerted a significant dual impact on nursing staff turnover intention: a substantial direct effect and a pronounced indirect effect mediated by occupational burnout. As the cornerstone of the healthcare system, the physical and mental well‐being of nursing personnel is intrinsically linked to the efficacy of public health emergency responses. Alarmingly, the escalating prevalence of burnout and PTSD among this critical workforce now represents a pressing systemic risk, demanding immediate and decisive intervention in both current and anticipated public health crises. To mitigate this imminent threat and preserve healthcare system resilience, we advocate the implementation of the following evidence‐based, actionable strategies: (1) delivering targeted interventions to alleviate PTSD symptoms; (2) optimising structural and operational working conditions; (3) instituting robust multilevel support systems. Critically, policy formulation and resource allocation must prioritise high‐risk subgroups, including male nurses, those with senior professional titles, individuals working extended hours and personnel in resource‐constrained grassroots healthcare settings. The prompt implementation of these feasible and targeted measures is imperative to enhance workforce stability, operational efficiency and the overall resilience of the healthcare system during public health emergencies.

## Author Contributions

J.Z. and X.M. conducting research, obtaining research funding, collecting data, analyzing and interpreting data, and writing papers; Y.Z. data collection, paper revision; J.G. data collection, administrative and technical support; Y.W. data collection, statistical analysis, and paper revision; L.W. data collection, paper revision and guidance; J.C. research design, obtaining research funding, paper revision and guidance.

## Funding

Research and Cultivation Program of Beijing Hospital Management Center (Grants PX2023068 and RMB60000); Project Name: Construction and Application of a Multimodal Exercise and Meditation Program for Patients with Depression and Insomnia. The Beijing High‐Level Innovation and Entrepreneurship Talent Support Program‐leading talent projects (Grant G202511067).

## Ethics Statement

This study complied with the Declaration of Helsinki and was approved by the Ethics Committee of Beijing HuiLongGuan Hospital. Approval unit: Beijing HuiLongGuan Hospital; Approval number: 2023‐23—Science and Technology.

## Conflicts of Interest

The authors declare no conflicts of interest.

## Data Availability

The data that support the findings of this study are available from the corresponding author upon request.
